# Diagnosis and management of treatment-refractory hypothyroidism: an expert consensus report

**DOI:** 10.1007/s40618-017-0706-y

**Published:** 2017-07-10

**Authors:** M. Centanni, S. Benvenga, I. Sachmechi

**Affiliations:** 1 Section of Endocrinology, Department of Medico-surgical Services and Biotechnologies, Sapienza University of Rome, Latina, Italy; 2 Section of Endocrinology, Department of Clinical and Experimental Medicine, University of Messina, Messina, Italy; 3 Interdepartmental Program of Molecular and Clinical Endocrinology and Women’s Endocrine Health, A.O.U. Policlinico G Martino, Messina, Italy; 4 Division of Endocrinology, Queens Hospital Center, Icahn School of Medicine, Jamaica, NY USA

**Keywords:** Hypothyroidism, Levothyroxine, Malabsorption, Refractory

## Abstract

There is a frequently encountered subset of hypothyroid patients who are refractory to standard thyroid hormone replacement treatment and require unexpectedly high doses of levothyroxine. In addition to clinical situations where hypothyroid patients are non-compliant, or where there is the possibility of excipient-induced disease exacerbation (gluten/celiac disease), therapeutic failure may be due to impaired absorption of the administered drug. The common approach to managing patients with unusual thyroxine needs is to escalate the dose of levothyroxine until targeted TSH levels are achieved. This approach can increase the risk for prolonged exposure to supratherapeutic doses of levothyroxine, which increase the chances of adverse outcomes. Repeated adjustments of levothyroxine can also escalate the costs of treatment, as frequent office visits and laboratory tests are required to determine and maintain the desired dose. Clinicians should take a systematic approach to managing patients whom they suspect of having treatment-refractory hypothyroidism. This may include searching for, and adjusting, occult medical conditions and/or other factors that may affect the absorption of levothyroxine, before up-titrating the dose of traditional levothyroxine therapy. Depending on the underlying pathology, another approach that may be considered is to try alternative formulations of levothyroxine that are less susceptible to intolerance issues related to excipients, or, in some cases, to malabsorption. The early discovery of these factors via a thoughtful patient work-up may avoid unnecessary thyroid medication adjustments and their consequences for both patients and clinicians.

## Introduction

Primary hypothyroidism is considered to be refractory to oral thyroxine when there is biochemical or clinical evidence of hypothyroidism [serum level of thyroid-stimulating hormone (TSH) above the upper target level, usually 4.5 mU/L following a 6-week interval after the dosage was last increased] and/or unresolved hypothyroid symptoms, despite increasing dosages of levothyroxine beyond 1.9 μg/kg daily [1–3]. Further increments in the dosage of thyroxine may not always be the most appropriate intervention as supratherapeutic doses have been associated with cardiovascular and other side effects. When confronted with cases in which unexpectedly high doses of thyroxine are required, clinicians should confirm compliance and search for causes of decreased absorption or increased demand for thyroxine (Table 1) [3–8]. This consensus paper provides current thinking on the causes of refractory hypothyroidism and recommendations for its diagnosis and management.

**Table 1:** Causes of treatment-refractory hypothyroidism

Decreased bioavailability
Poor adherence to, or tolerability of, drug therapy
Maldigestion due to patient-related factors or behavior
Proton-pump inhibitor therapy
Gastric infection with *Helicobacter pylori*
Intestinal malabsorption of l-thyroxine
Luminal factors (e.g., food, coffee, and medications)
Intramural factors (e.g., short bowel syndrome, lactose intolerance, gluten enteropathy, inflammatory bowel disease, infiltrative enteropathy, infection with *Giardia*)
Increased need for levothyroxine
Weight gain
Pregnancy
Increased metabolism of thyroxine
Other factors that can alter serum levels of TSH
Addison’s disease
Altered regulation of the hypothalamic-pituitary-thyroid axis
TSH heterophile antibodies
Inappropriate tablet storage

*TSH* thyroid-stimulating hormone [3–6, 8]

## Background

Refractory hypothyroidism is a clinical condition that is increasingly recognized worldwide [8–10]. Even though its prevalence has not been precisely documented, non-optimal thyroxine therapy, as evidenced by TSH levels above (or below) the reference range, is common (Table 2) [11–17]. The most recent data supporting this finding were reported by Vaisman in a multicenter study from Brazil, in which 28% of 2292 hypothyroid patients had elevated serum TSH levels with a background of thyroid hormone therapy [15]. Results from prior trials are consistent with this finding and are suggestive that a substantial percentage of patients may not achieve their TSH target when using levothyroxine [11–14]. Since levothyroxine is the first-line treatment for hypothyroidism [18, 19] and one of the most widely used drugs in the US with more than 115 million prescriptions dispensed in 2013 [20], a fresh examination of the causes of its frequent sub-optimal performance is warranted.

**Table 2:** Results from five published studies measuring TSH levels

	Optimal thyroxine therapy (%)	Non-optimal therapy^a^ (%)
Ross [11]	68	32
Parle [12]	52	48
Canaris [13]	60	40
Hollowell [14]	67	33
Vaisman [15]	58	42

^a^Inadequate thyroxine therapy: 18, 27, 18, 15, 28%; Excessive thyroxine therapy: 14, 21, 22, 18, 14% [11–15]

## Refractory hypothyroidism—clinical and laboratory findings

Diagnosis of refractory hypothyroidism is both art and science. The traditional diagnostic tools such as laboratory tests (TSH, free (F) T4, free (F) T3, and others) can indicate thyroid dysfunction. However, it is not uncommon to discover symptoms of both hypo- and hyperthyroidism in the context of diagnostic findings that are within the desired reference range specified by the current treatment guidelines [18]. Supratherapeutic doses of levothyroxine, which can induce iatrogenic hyperthyroidism, should not be used as a strategy to suppress hypothyroidism symptoms. In the panel’s experience, such patients may be referred to a specialist for additional evaluation after having been initiated on unexpectedly high doses of levothyroxine (>1.9 µg/kg/day) and continuing to experience persistent symptoms of hypothyroidism.

### TSH testing

According to the most recent guidelines from the American Thyroid Association (ATA), “TSH is the most reliable marker of adequacy of replacement treatment, and a value within the reference range (0.4–4.0 mIU/L) should be considered the therapeutic target” [18]. Subclinical hypothyroidism is generally characterized by a serum TSH level above the upper reference limit, combined with a normal FT4. This definition is applicable only when thyroid function has been stable for many weeks, there is a normal hypothalamic–pituitary–thyroid axis, and no recent or ongoing severe illness. Overt hypothyroidism is typically characterized by an elevated TSH (above 10 mIU/L) in combination with a subnormal FT4 [21].

In the appropriate clinical context, a subnormal serum FT4 level usually establishes the diagnosis of hypothyroidism, whether primary (elevated serum TSH) or central (normal or low serum TSH) [22, 23]. The primary test for detecting hypothyroidism in patients with secondary hypothyroidism who are on a stable regimen of levothyroxine is a serum FT4 assessment. FT4 is also the primary measurement in patients who have been recently started on antithyroid drugs or have recently undergone surgical/radioiodine ablation and were previously diagnosed as hyperthyroid based on low serum TSH levels over many weeks or months [21].

### FT3 testing

As is the case with T4, T3 is also bound to serum proteins, principally T4-binding globulin (TBG), but to a lesser degree than T4. Methods for determining T3 concentration by direct immunoassay are currently in use [21, 24]. FT3 measurement has somewhat limited utility, however, in the diagnosis of hypothyroidism, since FT3 values often fall in the normal range as a result of hyperstimulation of the remaining functioning thyroid tissue by elevated TSH and to up-regulation of deiodinase D2, the enzyme that converts T4 to T3 [25]. In the absence of thyroid disease in severely ill patients, FT3 levels may be low because of reduced peripheral conversion of T4 to T3 and increased thyroid hormone inactivation [21, 26, 27].

### TSH vs. FT3 testing

It is important to note that TSH is the most sensitive target gene of thyroid hormones. Even minimal changes in the content of thyroid hormones in the thyrotrophs can affect the synthesis of serum TSH and its secretion into the bloodstream. As reported by Taylor and colleagues [28], although a modest increase in the content of T4 (and T4-derived T3) found in the thyrotrophs will usually cause a substantial reduction in serum TSH in the setting of hypothyroidism, the same modest level of increase in peripheral tissues (liver, muscles, etc.) does not cause appreciable changes in target genes. Clinically speaking, a decline in serum TSH to normal levels (pituitary euthyroidism) can, in theory, still coexist with tissue hypothyroidism in one or more target tissues. Residual hypothyroid symptoms may be a consequence of insufficient hormone levels in some peripheral tissues.

A lack of correlation between TSH and peripheral tissue levels of T3 is worsened in conditions such as chronic emotional or physical stress, chronic illness, diabetes, insulin resistance, obesity, leptin resistance, depression, chronic fatigue syndrome, fibromyalgia, premenstrual syndrome (PMS), and both dieting or weight gain. In such conditions, tissue levels of T3 are shown to drop dramatically out of proportion with serum T3 [29–37]. While serum T3 levels may decline by 30%, which is significant, but still within the so-called “normal range”, tissue T3 may decline by 70–80%, resulting in profound cellular hypothyroidism with normal serum TSH, FT4, and FT3 levels [29–39]. As a result, TSH may be a poor indicator of peripheral thyroid levels in the presence of such conditions. In addition, a normal TSH should not always be considered a reliable indicator for euthyreosis, especially in patients presenting with symptoms consistent with thyroid deficiency [40]. Several conditions such as age, pregnancy, and the presence of thyroid hormone antibodies may complicate the interpretation of TSH values [6, 8, 18]. In spite of these limitations, TSH remains the best means of assessing thyroid function in over 80% of patients.

## Primary factors contributing to refractory hypothyroidism

In cases where TSH levels are unable to be maintained within a desired TSH range without the use of unexpectedly large daily doses of thyroxine therapy, clinicians should suspect the presence of one or more common conditions that can make patients refractory to the traditional thyroid hormone treatment.

### Non-pathologic causes of refractory hypothyroidism

1. *Non-compliance* For many years, poor compliance with the daily dosing of levothyroxine was reputed to be the most common reason for unusually high doses of thyroid replacement therapy [1]. Because of its long half-life, missing 1 day of levothyroxine therapy has an influence on thyroid hormone and TSH levels that can extend for several days [41].
  The problem of non-compliance with thyroxine therapy has traditionally been viewed within the context of missed doses of therapy. However, in the recently published CONTROL Surveillance Project among 925 patients on levothyroxine therapy, McMillan et al. reported that many patients do not take thyroxine therapy as indicated. More than 21% reported taking thyroxine less than the recommended 30 min before eating, a practice that may significantly contribute to less-than-optimal drug absorption [42].
2. *Switching to a generic levothyroxine with different bioavailability* Despite considerable efforts and progress by the healthcare community and regulatory bodies, concerns over levothyroxine substitution persist. Interchange of levothyroxine preparations at the pharmacy has been shown to contribute to sub-optimal management of hypothyroidism. In 2010, the American Association of Clinical Endocrinologists (AACE), American Thyroid Association (ATA), and The Endocrine Society (TES) collaborated to conduct a survey of their society members and frequent prescribers of levothyroxine. From more than 18,000 emailed requests for information, the investigators found that the clinical use of contemporary levothyroxine products continues to be associated with adverse outcomes. The adverse outcomes most frequently reported were those associated with the generic substitution of levothyroxine products, frequently without the knowledge of the prescribing physician. Most of the cases resulted in either mild symptoms of hypo-or hyperthyroidism and/or unexpected thyroid function tests that were outside normal limits [43].
3. *Drugs that treat GI conditions/dietary considerations* In tablet formulations, levothyroxine is absorbed primarily at the jejunum and upper ileum. Several non-pathologic factors that have been shown to affect levothyroxine absorption and performance include diet (timing of ingestion relative to meals and beverages, including coffee), and use of certain nutritional supplements, vitamins (including vitamin D), and medications such as proton-pump inhibitors (PPIs), histamine receptor blockers, cholestyramine, and motility modifying agents. Sensitivity to ingredients contained in medication, poor tolerability, and, occasionally, improper storage of tablets have been shown to contribute to levothyroxine malabsorption (Table 1) [3, 8, 44–59].
  In the CONTROL Surveillance Project, McMillan et al. documented the prevalence of factors that can affect the absorption and tolerability of levothyroxine. These included the presence of comorbid conditions such as gastroesophageal reflux disease (33.8%), irritable bowel syndrome (IBS) (9.7%), lactose intolerance (7.8%), or the use of prescription and OTC medications used to treat these conditions (34%). The use of dietary supplements (most notably iron and calcium) was reported in almost 52% of patients. Intake of foods and beverages high in fiber, iodine, or soy was reported in 68% of patients. More than 15% of patients reported allergies to ingredients that are known to be included in most tablet drug formulations [42].
4. *Pregnancy* Among patients undergoing body mass changes in the context of pregnancy, additional factors need to be considered. Alexander et al. prospectively studied 20 pregnant women with treated hypothyroidism. Early in the first trimester, the investigators noted an increased levothyroxine requirement of up to 50%, which peaked midway through pregnancy and remained constant until delivery [60]. Women with treated hypothyroidism, accordingly, may need to increase their dose of levothyroxine to prevent hypothyroidism and its attendant adverse outcomes for pregnancy [61]. A pregnancy test should be considered for women of reproductive age suspected of having treatment-refractory hypothyroidism.
  The most recent ATA guidelines provide the following trimester-specific reference ranges for TSH: “The treatment of hypothyroidism during pregnancy must be considered within the context of trimester-specific alterations in thyroid physiology as well as the etiology of the thyroid disease. The TSH range for each trimester should be defined within the medical system in which care is being provided, with a generalized range as follows: 0.1–2.5 mIU/L for the first trimester, 0.2–3.0 mIU/L for the second trimester, and 0.3–3.0 mIU/L for the third trimester” [18].

### Pathologic causes of refractory hypothyroidism

1. *Concomitant GI diseases* Possibly misunderstood in the past, or even confused with pseudomalabsorption [62], malabsorption of levothyroxine is now widely recognized as a legitimate medical problem in a significant percentage of hypothyroid patients. Levothyroxine absorption can be limited by a number of diseases of the gastrointestinal (GI) tract, including: *Helicobacter pylori* infection, inflammatory bowel disease (IBD), celiac disease, lactose intolerance, atrophic body gastritis (ABG), gastric bypass, biliary pancreatic diversion, and gastroparesis, among others [5, 10, 17, 52, 63]. The presence of any of these conditions may adversely affect the absorption of levothyroxine and its dose requirements [3, 9, 17, 47, 49, 53, 54, 57, 64–70]. Collectively, these widespread disorders represent the most important causes of increased need for thyroxine.
2. *Changes in body weight/body mass* An important factor contributing to increased demand of levothyroxine is weight change. Although the total daily dose of levothyroxine is usually higher in individuals who are obese, the dose per kilogram tends to be lower. In a case series of 75 consecutive post-thyroidectomy patients, an inverse relationship between the dose of levothyroxine required to normalize TSH levels and body weight was reported [71]. In a prospective study involving 100 post-thyroidectomy patients, the optimal dose of levothyroxine (µg) could be predicted using a simple formula: dose = weight (kg) − age (years) + 125 [72]. It is important to note that lean body mass is a better standard to gauge thyroxine requirements than weight.
  Rapid weight loss due to serious illness (e.g., certain malignancies, AIDS, malnutrition, and bariatric surgery) has also been associated with oral levothyroxine homeostasis [73].
3. *Poor conversion of T4 to T3* It has been suggested that a genetic variation in deiodinase D2 (also called type 2 deiodinase, or 5′-deiodinase) may help to explain why some patients who fail to achieve control of hypothyroidism with a background of levothyroxine therapy respond to natural desiccated thyroid or to the combined therapy of levothyroxine and synthetic T3 [74]. In a 2011 study, Gullo et al. concluded that while the long-term effects of chronic tissue exposure to an abnormal T3/T4 ratio are not known, a sensitive marker of target organ response to thyroid hormones (i.e., serum TSH), suggests that the need for combination T3/T4 therapy may stem from abnormal pituitary response. Thus, some patients may require a more “physiological” treatment than levothyroxine monotherapy [75].
  Kim and Bianco [76] postulated that some thyroid patients have a less effective deiodinase D2 enzyme for the conversion of T4 to T3. The authors suggest that this alteration occurs in 16% of the “poor converters” studied, and conclude that the majority of hypothyroid patients do not have this problem and can achieve the desired therapeutic response on T4 monotherapy. Bowthorpe et al. have suggested that these “poor absorbers” need combined therapy of T4 and T3 [74]. However, the benefits of that treatment remain controversial [77, 78].

## Less common causes of refractory hypothyroidism

Other causes of treatment-refractory hypothyroidism are usually more difficult to identify. These include dysfunction in the hypothalamic–pituitary–thyroid axis (resistance to thyroid hormone) and Addison’s disease [8]. Benvenga and others have reported the occurrence of hypothyroidism due to levothyroxine malabsorption in patients with cystic fibrosis [9, 79–81]. Cystic fibrosis can be complicated by amyloidosis [82, 83] that can have a direct effect on the thyroid [9, 84]. In 2009, Morris discussed nephrotic syndrome as another potential cause of elevated TSH levels [41, 85–87]. Patients with autoimmune thyroid disease may be at higher risk of developing resistance to the traditional thyroid hormone replacement therapy. In a recent study, Yamamoto et al. demonstrated the prevalence of anti-thyroxine antibodies among 187 patients with autoimmune thyroid diseases. Among those patients, such antibodies were discovered in 17.1% of patients (32/187) vs. 3.4% of patients (2/58) with non-autoimmune thyroid diseases [88].

## Unknown reasons for refractory hypothyroidism

Suzuki and colleagues reported failure to identify a cause in approximately 10–20% of patients requiring greater than standard replacement doses of levothyroxine despite an extensive diagnostic work-up. Such patients have always existed [89]. Benvenga and Centanni have also reported rates of 10–20% for apparent idiopathic refractory hypothyroidism [9]. Hays described “thyroid-resistant” patients in a paper published in 1968. In that early study, Hays estimated intestinal absorption of thyroxine from the serum ^125^I/^131^I ratio after simultaneous administration of oral thyroxine-^125^I and intravenous thyroxine-^131^I. Hays characterized as “thyroid-resistant” four patients who were studied specifically because they appeared, clinically, to require, or at least tolerate, unusually high doses of thyroxine. All showed normal absorption percentages. Hays noted that the possibility that these patients may have had difficulty dissolving the usual tablet dosage forms of thyroxine was not likely. This study did show, however, that other patients with clinical malabsorption of food products do absorb thyroxine poorly. Whether or not this finding turns out to be clinically important, Hays further noted, remains to be seen [90].

These patients are frequently managed with parenteral administration of levothyroxine [9, 91–93]. Recently, a possible role for an altered intestinal microbiota composition has also been conceived [94, 95]. Our specific recommendations for diagnosing and managing refractory hypothyroidism resulting from the wide range of etiologies discussed in this paper are presented below.

## Discussion

As noted above, primary hypothyroidism is considered to be refractory to oral thyroxine when there is biochemical or clinical evidence of hypothyroidism (serum level of TSH above the upper target level, usually 4.5 mU/L following a 6-week interval after the dosage was last increased) and/or unresolved hypothyroid symptoms, despite increasing dosages beyond 1.9 μg/kg daily [1–3]. The most common approach to managing such patients is to escalate the dose of levothyroxine or change the levothyroxine formulation until target TSH levels are achieved and hypothyroid symptoms are controlled. However, due to the narrow therapeutic index of levothyroxine, individualized dose titration can sometimes require considerable trial and error.

There are several drawbacks to this approach, including:

1. *Effects on bone integrity* Evidence in the clinical literature suggests that there is an increased risk of bone fracture in individuals who are exposed to supratherapeutic doses of exogenous thyroid hormone for extended periods of time. According to Bauer et al. the risk of fracture is related to the degree of TSH suppression (TSH <0.1 mU/L vs. TSH 0.1–0.5 mU/L) in addition to other factors such as age [96]. In a recent observational cohort study that examined the risk of fracture in 17,684 patients >18 years old (mean age: 60.3 females, 61.8 males) on long-term levothyroxine therapy, a twofold increase in fracture risk was reported in patients having undetectable TSH levels (≤0.03 mU/L) compared to those with TSH levels in the normal range [97]. More recently, Abrahamsen and colleagues found the long-term risk of hip and other osteoporotic fractures among real-world patients to be strongly correlated with the cumulative duration of periods with low TSH—most likely a consequence of excessive replacement [98].
  While it is generally accepted that lengthy periods of hyperthyroidism affect bone mass density (BMD) and can increase the risk of osteoporosis, there is significant controversy as to whether the long-term use of levothyroxine at suppressive or non-suppressive levels actually increases osteoporosis risk. Comprehensive, definitive research is required to establish with certainty whether suppressed or low-normal TSH levels increase the risk of osteoporosis and/or fractures, and whether the use of supplemental T3 has any effect at all on the risk of osteoporosis. The extent of such risk should also be assessed in comparison to the large number of other health risks (including the very real risk of heart disease) associated with elevated TSH levels or physiologic hypothyroidism [99]. The first-line treatment of hypothyroidism dictates that thyroxine dosing be carefully monitored and adjusted as necessary to prevent ongoing bone loss [100].
2. *Cardiovascular and other effects* Excessive thyroid hormone has been associated with an increase in cardiovascular conditions such as tachycardia, left ventricular hypertrophy, and poor diastolic relaxation. Such individuals are usually uncomfortable, and their pulse is high. Simply lowering the dose of levothyroxine often solves the problem. Unfortunately, cardiac issues can develop, even in the absence of excessive thyroxine dosing in susceptible individuals [101]. In an observational cohort study, using data linkage from regional data sets between 1993 and 2001, Flynn and colleagues studied the safety of 17,684 patients with a low, but not suppressed, serum TSH when receiving long-term replacement therapy with levothyroxine. The researchers concluded that there was an increased risk of cardiovascular disease, dysrhythmias, and fractures in patients having high or suppressed TSH, but not in patients with low, but unsuppressed, TSH levels [97].
  As noted above, the potential consequences of supratherapeutic doses of levothyroxine are well known. As a result, many clinicians choose to start patients, especially those who may be at risk for ischemic heart disease, on low doses of levothyroxine and titrate up slowly until an ideal dose is achieved. Some patients are maintained permanently on sub-therapeutic or minimal doses of levothyroxine out of an abundance of concern. In our experience, this approach is flawed. Patients kept on sub-therapeutic doses of levothyroxine for long periods not only may suffer the classic symptoms of uncontrolled hypothyroidism, but may experience other significant physiologic effects. In a recent study, Piantanida et al. demonstrated a correlation between uncontrolled hypothyroidism and increased risk for masked hypertension (MH). Masked hypertension, or the association of normal office blood pressure readings with high ambulatory or home measurements, is a significant risk factor for target organ damage. The authors concluded that restoration of euthyroidism via thyroid replacement therapy can help lower blood pressure and cardiovascular risk among patients with MH [102]. Similar results were noted by Gallo et al. in a study measuring neuromuscular symptoms in 57 newly diagnosed hypothyroid patients. Among those patients with overt hypothyroidism, there was a significantly higher prevalence of both neuromuscular symptoms (myalgias, slowness of movement, and tiredness) and elevated serum creatine phosphokinase (CPK) levels compared with euthyroid patients (*P* < 0.0001). After thyroid replacement therapy was initiated, normalization of CPK levels and restoration of normal neuromuscular function were observed. The authors concluded that thyroid hormone replacement therapy may be helpful in reversing abnormalities of physical performance which may have a negative impact on patient well-being and quality of life [103].
3. *Increased use of healthcare resources* Dose titration of levothyroxine is common in clinical practice and can result in unnecessary use of healthcare and other societal resources. In CONTROL HE, a retrospective study of 454 patients receiving levothyroxine for greater than 1 year, Ernst et al. reported a significant relationship between the number of levothyroxine dose or formulation changes and increased cost of patient care including drug therapy, laboratory tests, physician visits, and other healthcare costs (*P* < 0.05). These dose changes were also associated with an increase in lost wages and work productivity. Patients with ≥1 levothyroxine dose or formulation change in a 24-month period experienced an increase in total resource utilization of US $2658 versus patients who had not experienced changes to their levothyroxine therapy. Patients with ≥3 levothyroxine dose changes in a 24-month period had costs that were 2.5 times greater than patients with no dose or formulation changes (US $8220 vs. US $3166). According to the authors, levothyroxine dose and formulation changes escalate the socioeconomic burden of hypothyroidism care by both increasing healthcare costs and decreasing work productivity [104].
4. *Patient satisfaction with therapy* Levothyroxine dose and formulation changes can have an impact on patient satisfaction with care. In CONTROL TS, a survey involving 300 hypothyroid patients taking levothyroxine for more than 1 year, individuals reporting ≥2 levothyroxine dose changes in the past 12 months were significantly less satisfied with their hypothyroid treatment than those reporting no dose changes. Those patients were less likely to feel that their thyroid medication was controlling their hypothyroid symptoms and were less enthusiastic about continuing their current medication regimen. Overall patient satisfaction with treatment was 44% lower among patients experiencing levothyroxine dose changes versus those without such changes (*P* < 0.001). Patients whose levothyroxine therapy had been changed multiple times were also more likely to be dissatisfied with their hypothyroid care and more likely to have changed their hypothyroid-treating physician in the past year: +66.1% (*P* = .054) [105].

## Diagnosing and managing refractory hypothyroidism

### Key principle

When confronted with patients in which escalating doses of thyroid replacement hormone have failed to control TSH levels and symptoms of hypothyroidism, clinicians should employ a systematic approach to gathering information and determining an effective therapeutic strategy. We recommend the following eight-step approach, which has been validated through empirical use and should be considered as an alternative to the standard practice of escalating doses of levothyroxine:

1. *Confirm the diagnosis and laboratory results* By laboratory definition, frank primary hypothyroidism requires low levels of thyroid hormones (free–FT4 and FT3) as well as elevated TSH. The finding of persistently elevated TSH is insufficient to confirm the diagnosis—it is also essential to measure thyroid hormone levels (FT4 and FT3). Markedly elevated TSH levels in the absence of low or at least low-normal thyroid hormones suggest other diagnoses or explanations for the increased dose requirements (e.g., heterophilic antibody interference with TSH measurements) [41]. It is important to remember that TSH levels are best interpreted in light of each patient’s clinical history (familial, pharmacological, surgical, other morbidities, etc.)
2. *Ask about compliance* Poor compliance is the most common reason for unusually high thyroid hormone dose requirements. Patients will often acknowledge occasionally forgetting their levothyroxine tablets if asked in a non-accusatory, non-judgmental manner. What can be difficult is determining how often ‘occasionally’ actually occurs [41]. If necessary, clinicians can investigate adherence to therapy by direct patient report, clinical impression, or frequency of pharmacy refills. A supervised test for levothyroxine absorption may be useful if poor adherence to oral treatment is suspected [8, 106, 107].
3. *Check the patient’s medication bottles and tablets* The patient’s reported dose may, at times, differ from that prescribed, and pharmacy errors can also occur, resulting in tablets inside the bottle that are not the same as those reported on the label [41]. In difficult-to-control hypothyroid patients, the panel also recommends checking for appropriate storage of levothyroxine tablets (e.g., protection from light, moisture, and temperature extremes, as stated in the package insert) [108].
4. *Review the thyroxine ingestion history* The most efficient way to take levothyroxine—and the most reproducible—is to ingest the tablets on an empty stomach and avoid other medications or food for 30–60 min (preferably 60) thereafter [4, 5, 48]. As noted above, a large and growing number of medications, supplements, and food items have been shown to alter the fraction of an ingested dose that is absorbed [109, 110]. The ingestion of one or more of these at, or close to, the time of levothyroxine dosing can significantly change the dose requirement in individual patients, especially when done regularly [49].
5. *Investigate for levothyroxine malabsorption* Inter-individual variability in the efficiency of GI absorption is rather large, and such variability accounts for most of the range of dose requirements seen among compliant patients following adjustment for body size. The first sign of malabsorption syndrome, which is frequently oligosymptomatic and previously undiagnosed, can be the need for unexpectedly high doses of levothyroxine in replacement therapy. Individuals taking levothyroxine in daily doses exceeding 1.9 μg/kg body weight, and who have persistently elevated TSH levels and/or unresolved symptoms of hypothyroidism, should be evaluated, and, if indicated, undergo testing for malabsorption. We are recommending a diagnostic flow chart (Fig. 1), which, based on increased thyroxine requirements [10], can help to establish the presence of concomitant GI disorders [111–113]. Frequently, correction of the malabsorption will normalize, or at least improve, levothyroxine absorption in patients with these disorders [10].

**Fig. 1:**
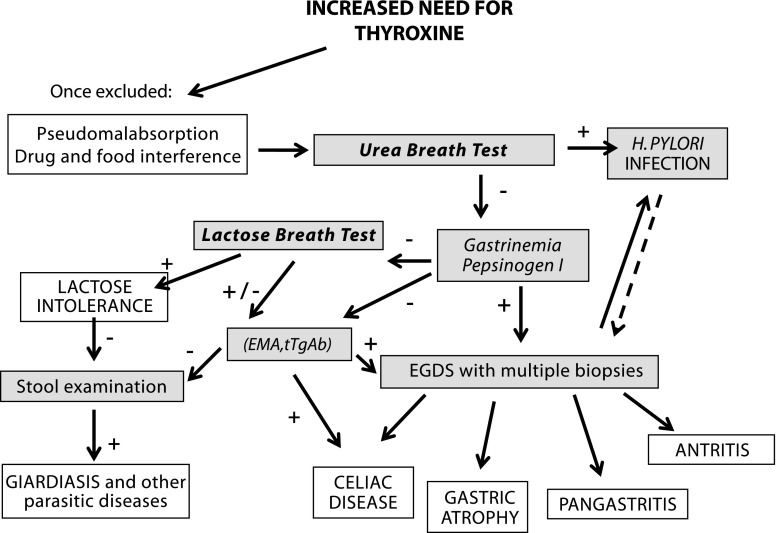
Diagnostic flow chart of thyroxine malabsorption. + if test is positive, − if test is negative. *EMA* endomysial antibody. (Modified from Centanni M, hotthyroidology.com 2007)
6. *Consider increased turnover or excretion* Several drugs or clinical conditions can increase the turnover or excretion of thyroid hormone, thereby substantially increasing the requirement in patients who are thyroid hormone dependent. These medications include phenytoin, carbamazepine, and rifampin. A number of kinase inhibitors, including imatinib and sunitinib, appear to influence thyroxine requirements through what is likely a class effect [114–116]. Moreover, patients with nephrotic syndrome who excrete large quantities of albumin may also have increased thyroxine requirements as a result of the binding of levothyroxine to the excreted albumin [87]. As mentioned above, during normal pregnancy, women experience increased thyroxine requirements that may approach 50% [41, 60].
*Perform a thyroxine absorption test* Although not standardized, a clinical test for estimating the absorption of thyroxine may have utility in some patients who have unexpectedly high thyroxine requirements. The absorption test involves administration of a single large dose of levothyroxine, typically in the range of 600–1000 μg, followed by monitoring serum thyroxine levels over time [110]. Using a high-dose levothyroxine, absorption test can play an important role in the differential diagnosis of pseudomalabsorption (which is a most common source of difficulties in obtaining euthyreosis in hypothyroid patients) and actual levothyroxine malabsorption [117, 118]. Because it is not standardized, and unlikely to be associated with a specific etiology, the absorption test should not be used routinely [9, 41, 51].
*Treat the patient holistically* If a mistake by the patient or a pharmacy-related cause is identified, correction of these factors may resolve the problem. In some situations, compliance issues can be treated best by increasing the size of the tablet. As a last resort in those with continuing poor compliance, administration of thyroxine once weekly can be successful [119]. When possible, removing interfering drugs (such as PPIs) or changing ingestion patterns can also be helpful. In select patients, weight control can be an important step in the right direction. Such individualized treatment can improve the overall cost of care.

### Alternative levothyroxine formulations

Another approach that may be considered is to try alternative formulations of levothyroxine that are less susceptible to intolerance or malabsorption. Two novel formulations of levothyroxine sodium are now available, a softgel and a liquid. The efficacy and tolerability of these alternative formulations of levothyroxine have recently been reported in the scientific literature [120].

In particular, Santaguida et al. [121] have shown that most patients with definite gastric disorders benefited by changing from the traditional tablet formulations to the softgel levothyroxine preparation at a reduced dose. CONTROL Switch, a recent retrospective study involving 99 hypothyroid patients being treated by endocrinologists, suggested that a strategy of switching therapy from the traditional levothyroxine tablets to levothyroxine gel caps may preclude or reduce the need for levothyroxine dose or formulation changes, while improving hypothyroid symptom control, in some patients [122]. In that study, switching primarily from either branded or generic levothyroxine tablets to levothyroxine gel caps resulted in:

- Significant reduction in dose adjustments; −55% (*P* < 0.0001). More than 85% of patients experienced ≤1 dose adjustment post-switch.
- Significant improvement in hypothyroid symptom control; +62% (*P* < 0.0001). Among patients switched for efficacy reasons (*n* = 25), 64% experienced improved symptom control.

As far as it concerns the liquid formulation, Fallahi et al. studied the effect of switching 152 patients from levothyroxine tablets to a liquid formulation of levothyroxine. Unlike prior studies that evaluated the switch of patients from tablets to a gel cap formulation, this study evaluated the effects of switching patients with no prior history of levothyroxine malabsorption, gastric disorders, or use of drugs that are known to interfere with levothyroxine absorption. Serum TSH, FT4, and FT3 were evaluated after 1–3 months (first control) and 5–7 months (second control) after the medication switch. Following the switch to the liquid formulation, TSH levels among patients significantly declined compared to the basal value at both the first control (*P* < 0.05) and the second control (*P* < 0.01). Levels of both FT4 and FT3 were not significantly changed [123].

In the end, the decision to use an alternative formulation of levothyroxine may be one of expediency. Recent evidence suggests that either the softgel or liquid alternatives may eliminate a number of factors that contribute to levothyroxine tablet failure and refractory hypothyroidism. They may also offer options to patients who are non-compliant, have difficulty swallowing medication, or decline treatment because of sensitivity to excipients commonly contained in most commercially available tablet formulations. Patients with impaired gastric pH and those with sensitivity to excipients found in the traditional levothyroxine tablets can stand to benefit from these alternative options.

## Conclusions

There is a frequently encountered subset of hypothyroid patients who are refractory to standard thyroid hormone replacement treatment and require unexpectedly high doses of levothyroxine or who have erratic control of hypothyroidism, alternating between over-replacement and under-replacement. In addition to clinical situations where hypothyroid patients are non-compliant, or where there is the possibility of excipient-induced disease exacerbation (gluten/celiac disease), therapeutic failure may be due to impaired absorption of the administered drug. This is often the result of either patient pathophysiology (GI disease), the effects of other therapeutic agents, or diet (timing of ingestion relative to meals and beverages, including coffee). Significant swings in weight may also cause treatment-refractory hypothyroidism. Switching to generic levothyroxine products has also been correlated with sub-optimal outcomes.

The common approach to managing patients with unusual thyroxine needs is to escalate the dose of levothyroxine until targeted TSH levels are achieved. This approach can increase the risk for prolonged exposure to supratherapeutic doses of levothyroxine which increase the chances of adverse outcomes. Studies have demonstrated that even intermittent oversuppression of TSH using levothyroxine can result in increased bone loss, cardiovascular, and other more serious medical conditions [97, 99, 101]. Repeated adjustments of levothyroxine can also escalate the costs of treatment, as frequent office visits and lab tests are required to determine and maintain the desired dose. These can also reduce patient satisfaction with hypothyroid treatment.

In our view, clinicians should take a systematic approach to managing patients whom they suspect of having treatment-refractory hypothyroidism. This may include searching for occult medical conditions, or other factors that may affect the absorption of levothyroxine, before up-titrating the dose of the traditional levothyroxine therapy. Depending on the underlying pathology, another approach that may be considered is to try alternative formulations of levothyroxine that are less susceptible to tolerance issues related to excipients or, in some cases, to malabsorption. In all cases, we advocate that the eight-step algorithm presented be used to carefully assess patient needs before treatment choices are made; such a strategy offers an alternative to reductionism as stated by Trimarchi in 2015 [124]. This holistic approach acknowledges that treatment-refractory hypothyroidism is a condition that exists within the context of other important factors—GI disease, concomitant drug use, diet, and patient habits—whose presence may adversely affect thyroid replacement therapy. The early discovery of these factors via a thoughtful patient work-up may avoid unnecessary thyroid medication adjustments and their consequences for patients, clinicians, and the healthcare system.
